# Neoadjuvant Treatment for Resectable and Borderline Resectable Pancreatic Cancer: Chemotherapy or Chemoradiotherapy?

**DOI:** 10.3389/fonc.2021.744161

**Published:** 2022-02-14

**Authors:** Eva Versteijne, Ignace H. J. T. de Hingh, Marjolein Y. V. Homs, Martijn P. W. Intven, Joost M. Klaase, Hjalmar C. van Santvoort, Judith de Vos-Geelen, Johanna W. Wilmink, Geertjan van Tienhoven

**Affiliations:** ^1^ Department of Radiation Oncology, Cancer Center Amsterdam, Amsterdam University Medical Center (UMC), University of Amsterdam, Amsterdam, Netherlands; ^2^ Department of Surgery, Catharina Hospital, Eindhoven and GROW—School for Oncology and Developmental Biology, Maastricht University, Maastricht, Netherlands; ^3^ Department Medical Oncology, Erasmus Medical Center (MC) Cancer Institute, Rotterdam, Netherlands; ^4^ Department of Radiation Oncology, University Medical Center Utrecht, Utrecht, Netherlands; ^5^ Department of Hepatopancreatobiliary Surgery and Liver Transplantation, University Medical Center Groningen, Groningen, Netherlands; ^6^ Department of Surgery, Regionaal Academisch Kankercentrum Utrecht (RAKU), St Antonius Hospital, Nieuwegein, Netherlands; ^7^ Department of Internal Medicine, Division of Medical Oncology, GROW—School for Oncology and Developmental Biology, Maastricht University Medical Center (UMC+), Maastricht, Netherlands; ^8^ Department of Medical Oncology, Cancer Center Amsterdam, Amsterdam University Medical Center (UMC), University of Amsterdam, Amsterdam, Netherlands

**Keywords:** resectable pancreatic cancer (RPC), borderline resectable pancreatic cancer (BRPC), chemotherapy, radiotherapy, neoadjuvant treatment

## Abstract

Worldwide, there is a shifting paradigm from immediate surgery with adjuvant treatment to a neoadjuvant approach for patients with resectable or borderline resectable pancreatic cancer (RPC or BRPC). Comparison of neoadjuvant and adjuvant studies is extremely difficult because of a great difference in patient selection. The evidence from randomized studies shows that overall survival by intention-to-treat improves after neoadjuvant gemcitabine-based chemoradiotherapy or chemotherapy (various regimens), as compared to immediate surgery followed by adjuvant chemotherapy. Radiotherapy appears to play an important role in mediating locoregional effects. Yet, since more effective chemotherapy regimens are currently available, in particular FOLFIRINOX and Gemcitabine/Nab-paclitaxel, these chemotherapy regimens should be investigated in future randomized trials combined with (stereotactic) radiotherapy to further improve outcomes of RPC and BRPC.

## Introduction

The majority of patients with pancreatic neoplasms are pancreatic ductal adenocarcinoma (PDAC), containing 90% of the cases. Other subtypes include for example acinar carcinoma, pancreatic blastoma, or neuroendocrine tumors (1). The incidence of pancreatic ductal adenocarcinoma (PDAC) continues to rise, with approximately 460,000 new cases per year and the seventh leading cause of cancer-related deaths worldwide (2–4). At diagnosis, only 20% of patients have resectable (RPC) or borderline-resectable disease (BRPC) while 80% of patients have irresectable tumors with or without distant metastases (2). There are several explanations for the fact that most patients are diagnosed with PDAC at a late stage; various symptoms associated with pancreatic cancer are non-specific such as weight loss, abdominal pain, and anorexia. In addition, due to the aggressive nature of the disease, early stages usually lack symptoms. Resectability, based on vascular involvement, is based on a multiphase computed tomography (CT) including non-contrast-enhanced, arterial, venous, and portal contrast phase axial scans. Magnetic resonance imaging (MRI) can help to identify cancers that may be poorly be visible on CT imaging. Positron emission tomography (PET) CT is not routine used for staging. The differentiation grade is related to the sensitivity of the PET-CT; medium- or well-differentiated pancreatic cancers tend to be negative on PET-CT. For patients in whom CT fails to identify a tumor mass or in whom pathology did not confirm pancreatic cancer or results are indeterminate, a PET-CT has a beneficial value, with higher specificity and accuracy compared to CT or MRI (5). Serum carbohydrate antigen (CA) 19-9 is a well-established biomarker for PDAC and, if initially high, useful in monitoring during treatment (1).

The main treatment of patients with RPC and BRPC is surgery, but up to 20%–25% of the patients who receive an exploratory laparotomy or laparoscopy do not get the intended tumor resection (6, 7). Even after surgical resection, there is a high likelihood of local or distant recurrence within 2 years. For this reason, most trials that were intended to improve outcome in patients with RPC and BRPC have focused on the efficacy of adjuvant and neoadjuvant treatment (8). During recent decades, investigation of the efficacy of neoadjuvant treatment has steadily increased. In this overview, we discuss the background of neoadjuvant treatment and the evidence based on randomized studies for neoadjuvant chemotherapy and neoadjuvant chemoradiotherapy in patients with RPC and BRPC. Potential future options for further improvement will be discussed.

## Multimodality Treatment

The efficacy of adjuvant chemotherapy has been investigated in various trials. Currently, it is regarded as the standard treatment for patients with RPC or BRPC after resection of the tumor, since it leads to an overall survival benefit compared to surgery alone (9–12). Adjuvant (modified) FOLFIRINOX is currently considered the standard adjuvant treatment for patients who are fit enough to receive this regimen, based on the French-Canadian trial published in 2018 (12). For patients not eligible for FOLFIRINOX, gemcitabine–capecitabine is preferred based on the ESPAC-4 trial (13). An important drawback of the adjuvant strategy is the observation that approximately 50% of patients do not receive the intended adjuvant chemotherapy because of surgical complications, poor performance status, or early recurrence (14). Moreover, of the patients starting adjuvant chemotherapy, not all of them complete the intended treatment schedule (15, 16). Therefore, it has been suggested that neoadjuvant treatment is preferred over adjuvant treatment, without increasing postoperative morbidity and mortality (17). In the first decade of the 21st century, many non-randomized single arm trials in patients with RPC and BRPC focused on the efficacy of neoadjuvant treatment (8). Since it is assumed that the majority of patients with RPC or BRPC already have occult metastasis at presentation, the aim of neoadjuvant treatment was to directly treat micro-metastases, in part by improving the chance that patients received the intended systemic treatment. In addition, it was hypothesized that neoadjuvant treatment results in a downstaging of the primary tumor, thereby improving the microscopically radical resection rate (R0), which is known to be of prognostic importance. Also, patients with very aggressive tumors, not responding to the neoadjuvant treatment, could be spared futile surgery. Lastly, neoadjuvant treatment could improve the patient condition before major oncological surgery by focusing on boundary conditions (18).

Comparison between the results of adjuvant and neoadjuvant studies is challenging, since adjuvant trials only include patients with “resected” pancreatic cancer, fit for adjuvant treatment without progression of disease. In contrast, neoadjuvant studies usually include all patients with “resectable” pancreatic cancer, based on diagnostic imaging, before any treatment is given. Hence, these populations are prognostic incongruent. As stated above, many patients with resectable pancreatic cancer do not undergo the intended resection or are not fit enough for adjuvant chemotherapy after resection. Hence, adjuvant studies include the 40%–50% of patients initially diagnosed with RPC or BRPC with the best prognosis, whereas neoadjuvant studies include the entire population of RPC and BRPC. As this will inevitably result in confounding by indication, outcomes between adjuvant and neoadjuvant trials are not comparable. In fact, the only scientifically way to randomly compare the effect of neoadjuvant and adjuvant treatment is by comparing the entire population of RPC and BRPC on the basis of intention-to-treat (ITT). That is, based on the moment of diagnosis, whether or not they received the intended treatment.

## Neoadjuvant Treatment

The outcomes by ITT of neoadjuvant treatment compared with those of adjuvant treatment in RPC and BRPC were previously analyzed in systematic reviews. One systematic review and meta-analysis published in 2018 reported the overall survival by ITT of 3,484 patients from 35 studies with RPC or BRPC who had been treated with immediate surgery or neoadjuvant treatment (19). The 1,738 patients treated with neoadjuvant treatment were found to have better pooled median overall survival, a higher R0 rate (87% versus 67%), and fewer pathological lymph nodes, as compared with the 1,746 patients treated with immediate surgery. The resection rate was lower after neoadjuvant treatment (66% versus 81%). The difference in pooled median overall survival was greater for the patients with BRPC, as compared to the patients with RPC (6.4 months versus 0.5 months difference, respectively). Notably, the neoadjuvant regimens were different between the studies, as were the criteria for resection (19). Importantly, 24 out of 35 studies (69%) used neoadjuvant chemoradiotherapy, and 26 out of 35 studies (74%) used gemcitabine in the neoadjuvant regimen (19). A suggested benefit of neoadjuvant treatment in RPC and BRPC was further confirmed by various reviews and meta-analyses since this review was conducted (20–22). For patients with BRPC, other reviews confirm the survival benefit of neoadjuvant treatment over immediate surgery (20, 23), and its role appears more affirmative than for patients with RPC. A possible explanation for this difference in effect is that the downstaging of the primary tumor may be more important in patients with BRPC, in whom vascular involvement, and therefore the risk of positive margins, is more prominent (24). These patients may also have a higher risk of developing metastases during neoadjuvant treatment and thus the patients whose tumors behave more aggressive are selected out before surgery (25). The efficacy of neoadjuvant treatment in RPC has not yet been established with three meta-analyses being performed since 2018 (26–28). These systematic reviews showed an increased R0 resection rate, fewer pathological lymph nodes, but no significant survival benefit in RPC patients treated with neoadjuvant treatment (26–28). One of these three meta-analyses showed a survival benefit of neoadjuvant treatment only in their per-protocol analysis (26).

Until recently, no evidence was available from randomized controlled trials (RCTs) investigating the efficacy of neoadjuvant treatment. Three phase II/III RCTs have investigated the efficacy of neoadjuvant chemotherapy over immediate surgery. The Japanese phase II/III trial of neoadjuvant gemcitabine and S-1 versus immediate surgery, which randomized 362 RPC patients, was presented only as a conference abstract in 2019, and demonstrated a significant advantage of neoadjuvant treatment (median overall survival 36.7 versus 26.6 months; *p* = 0.015) (29). The phase II trial of Reni et al. randomized 88 patients to receive surgery followed by adjuvant gemcitabine, or surgery followed by six cycles of adjuvant PEXG (cisplatin, epirubicin, gemcitabine, and capecitabine), or three cycles of PEXG neoadjuvant and three cycles adjuvant. The median overall survival was 20.4 months after surgery and adjuvant gemcitabine, versus 26.4 months in the adjuvant PEXG group, and 38.2 months in the neoadjuvant group. This study did not continue to phase III trial, because new, more effective chemotherapy regimens were developed (30). The multicenter randomized phase II/III NEPAFOX trial was stopped after 40 patients due to poor accrual. In this trial, patients with RPC and BRPC were randomized between immediate surgery followed by adjuvant gemcitabine for 6 months or perioperative FOLFIRINOX. The median OS was 25.7 months in the immediate surgery arm versus 10 months in the perioperative FOLFIRINOX arm, but the patient numbers are too low to draw firm conclusions (31). The weighted median overall survival of these three RCTs, calculated with the formula of Gillen et al. (32), is 30.3 months for the patients treated with neoadjuvant chemotherapy, compared to 25.7 months in patients treated with immediate surgery (**Table 1**).

**Table 1:** RCTs investigating neoadjuvant chemotherapy versus immediate surgery.

Trial	Resectability	Number of patients	Treatment arms	Median OS neoadjuvant group	Median OS immediate surgery group
PACT-15 (30)	R	88	PEXG + Surgery **versus** Surgery + adjuvant gemcitabine **versus** Surgery + adjuvant PEXG	38.2	20.4/26.4
Prep-02/JSAP-05 (29)	R	362	Gemcitabine + S1 + Surgery + adjuvant S1 **versus** Surgery + adjuvant S1	36.7	26.6
NEPAFOX (31)	R/BR	40	FOLFIRINOX + Surgery + adjuvant FOLFIRINOX **versus** Surgery + adjuvant gemcitabine	10	25.7
**Total Number of patients**				233	257
Weighted median OS (months)*				30.3	25.7

OS, overall survival; PEXG, cisplatin, epirubicin, capecitabine, gemcitabine; S1, Teysuno; R, resectable; BR, borderline resectable; FOLFIRINOX, oxaliplatin, irinotecan, fluorouracil, and leucovorin.

*Weighted median OS derived by the formula of Gillen and colleagues in a previous systematic review (32): $m_{p}={\left(\sum_{i=1}^{k}\frac{wi}{m_{i}}\right)}^{-1}$ where m_i_ denotes the median survival in a study population i (with i ranging from 1 to k, where k is the number of included studies) and wi refers to a study-specific weight function.

In addition to the RCTs investigating the efficacy of neoadjuvant chemotherapy, five RCTs have investigated the efficacy of neoadjuvant chemoradiotherapy. Before 2019, two RCTs, started in 2003 and 2007, were both prematurely closed due to low accrual (33, 34). The NEOPA trial, of which the trial protocol was published in 2014, randomized patients with RPC and BRPC between neoadjuvant chemoradiotherapy (gemcitabine combined with 50.4 Gy radiotherapy) and immediate surgery. This trial was terminated prematurely because of slow accrual, and no results were presented (35). In 2018, the interim analysis of a Korean randomized phase II trial showed that compared to immediate surgery, neoadjuvant chemoradiotherapy (gemcitabine combined with 54 Gy radiotherapy) provided oncological benefits in patients with BRPC (median overall survival 21.0 versus 12.0 months; HR 1.97; *p* = 0.028) (36). Because of these positive results, the accrual was prematurely closed after accruing 50 eligible patients (36). In 2020, the results of the PREOPANC trial were published (37). The initial analysis failed to show a significant survival benefit, but secondary endpoints and subset analyses were in favor of the neoadjuvant approach, particularly for the patients with BRPC (37). The PREOPANC was the first phase III trial that reached full accrual on the efficacy of neoadjuvant chemoradiotherapy in RPC and BPRC (37). In this trial, the neoadjuvant chemotherapy consisted of full-dose gemcitabine, with the rationale that the chemotherapy dose in earlier studies was considered too low accounting for the large proportions of early systemic failures. The scheme of full-dose gemcitabine combined with hypofractionated radiotherapy was tested earlier in two phase II trials (38, 39). The PREOPANC trial randomized 246 patients with RPC or BRPC between neoadjuvant chemoradiotherapy and immediate surgery, both followed by adjuvant chemotherapy. In June 2021, the updated results were presented at the ASCO annual meeting (40). With a median follow-up of 59 months, a significant benefit in overall survival was found in patients treated with neoadjuvant treatment, with a 5-year overall survival (ITT) of 20.5% versus 6.5% in the patients treated with immediate surgery. Similar to the initial analysis, the secondary outcomes were in favor of the neoadjuvant treatment. These long-term results showed a significant improvement in overall survival for both patients with RPC and BRPC. A difference in treatment effect between these subsets could not be demonstrated (interaction test *p* = 0.56), suggesting a similar effect for both subsets with long-term follow-up. The weighted median overall survival of the RCTs investigating neoadjuvant chemoradiotherapy, calculated with the formula of Gillen et al., was 17 months after neoadjuvant chemoradiotherapy, compared with 14.4 months in patients treated with immediate surgery (**Table 2**).

**Table 2:** RCTs on efficacy of neoadjuvant chemoradiotherapy versus immediate surgery.

Trial	Resectability	Number of patients	Treatment arms	Median OS neoadjuvant group	Median OS immediate surgery group
Jang (36)	BR	58	CRT (Gemcitabine + 54 Gy RT) + Surgery **versus** Surgery + adjuvant CRT (Gemcitabine + 54 Gy RT)	21.0	12.0
Casadei (34)	R	38	Gemcitabine + CRT (54 Gy RT + Gemcitabine) + Surgery + adjuvant gemcitabine **versus** Surgery + adjuvant gemcitabine	22.4	19.5
Golcher (33)	R	66	CRT (Gemcitabine/cisplatin + 55.8 Gy RT) + Surgery + adjuvant gemcitabine **versus** Surgery + adjuvant gemcitabine	17.4	14.4
PREOPANC (40)	BR/R	246	Gemcitabine + CRT (Gemcitabine + 36 Gy RT) + Surgery + adjuvant Gemcitabine **versus** Surgery + adjuvant Gemcitabine	15.7	14.3
**Total Number of patients**				197	203
Weighted median OS (months)*				17.0	14.4

OS, overall survival; RT, radiotherapy; BR, borderline resectable; CRT, chemoradiotherapy; R, resectable; Gy, Gray.

*Weighted median OS derived by the formula of Gillen and colleagues in a previous systematic review (32): $m_{p}={\left(\sum_{i=1}^{k}\frac{wi}{m_{i}}\right)}^{-1}$ where m_i_ denotes the median survival in a study population i (with i ranging from 1 to k, where k is the number of included studies) and wi refers to a study-specific weight function.

In the recently published abstract of the ESPAC-5F trial, patients were randomized between three different neoadjuvant approaches or immediate surgery. The three different neoadjuvant approaches consisted of gemcitabine/capecitabine versus FOLFIRINOX versus chemoradiotherapy (capecitabine with 50.4 Gy radiotherapy). This trial demonstrated a significant survival benefit for the neoadjuvant approach over the immediate surgery group (HR 0.27, *p* < 0.001). Of the different neoadjuvant treatments, the 12-month survival estimate was 84% for the patients treated with FOLFIRINOX, compared with 79% in the gemcitabine/capecitabine group, 64% in the chemoradiotherapy group, and 42% in the patients treated by immediate surgery (41). The median follow-up was only 12 months and longer follow-up is awaited.

The random effect meta-analysis of six RCTs conducted on neoadjuvant treatment versus immediate surgery that published its final results so far compared the results of 850 patients with RPC or BRPC randomized to either neoadjuvant treatment (chemotherapy or chemoradiotherapy) or immediate surgery (42). On the basis of ITT, an improvement of 6 months in median overall survival was found in patients treated with neoadjuvant treatment, from 19.4 to 25.4 months. In addition, improvements in R0 resection rate and lymph node positivity were found after neoadjuvant treatment, without a significant difference in overall resection rate (42). Four of these RCTs used neoadjuvant chemoradiotherapy, and the results were independent of the type of neoadjuvant treatment. This meta-analysis represents the strongest evidence to date favoring neoadjuvant treatment in the management of RPC or BRPC. However, it should be kept in mind that the criteria for resection being used between these studies was slightly different. Recently, in addition to this meta-analysis, pooled data from three randomized trials were published, comparing neoadjuvant chemo(radio)therapy with immediate surgery in patients with RPC. The median disease-free survival was significantly longer, but the OS did not reach statistical significance in patients treated with neoadjuvant treatment. They concluded that the neoadjuvant approach may become a “standard of care” for all patients with RPC and recommended to focus on which of the available neoadjuvant therapies is the best (43).

To conclude, there is currently a shifting paradigm worldwide, from immediate surgery with adjuvant treatment, to a neoadjuvant approach. The clinical evidence for neoadjuvant treatment is mostly based on the long-term results of the PREOPANC trial, which shows together with the earlier RCTs presented in the meta-analyses, an improvement in overall survival both in patients with RPC and with BRPC (40, 42).

### New Developments in Neoadjuvant Chemotherapy

New chemotherapeutic regimens became available during accrual of the above-mentioned randomized studies. The trials providing the evidence for the neoadjuvant approach started before the era of FOLFIRINOX and Gemcitabine/Nab-paclitaxel and were mainly gemcitabine-based schedules that are currently considered old fashioned. Therefore, the optimal chemotherapy regimen for neoadjuvant treatment has not yet been established. In reviews, the use of FOLFIRINOX in the neoadjuvant setting has shown its benefit in patients with BRPC and locally advanced pancreatic cancer (44). As a result, FOLFIRINOX is often advised for young and physically fit patients and Gemcitabine/Nab-paclitaxel for more fragile patients. The large systematic review and meta-analysis on neoadjuvant FOLFIRINOX in patients with BRPC showed a patient-level-based median overall survival of 22.2 months (45).

The recently published abstract of the SWOG S1505 trial, comparing neoadjuvant modified FOLFIRINOX with neoadjuvant Gemcitabine/Nab-paclitaxel for patients with RPC, did not find a significant difference in survival between the two treatments (median overall 22.4 months versus 23.6 months) (46). The final manuscript is not yet published. Currently, at least five other randomized trials on neoadjuvant chemotherapy are ongoing (47–50). Of these, three phase II/III trials are recruiting patients and investigating the efficacy of neoadjuvant FOLFIRNOX in patients with RPC (**Table 3**) (49, 50, 52). In addition, the PREOPANC-3 trial will investigate the efficacy of neoadjuvant FOLFIRINOX versus adjuvant FOLFIRINOX in patients with RPC, and is open for accrual.

**Table 3:** Ongoing RCTs on efficacy of neoadjuvant chemotherapy.

Trial	Resectability	Number of patients	Treatment arms	Status
NEONAX (48)	R	166	Gemcitabine/Nab-paclitaxel + Surgery + adjuvant Gemcitabine/Nab-paclitaxel **versus** Surgery + adjuvant Gemcitabine/Nab-paclitaxel	Recruiting
NEOPAC (47)	R	310	Gemcitabine + Oxaliplatin + Surgery + adjuvant Gemcitabine **versus** Surgery + adjuvant Gemcitabine	Completed
NORPACT-1 (49)	R	90	FOLFIRINOX + Surgery + adjuvant Gemcitabine/Capecitabine **versus** Surgery + adjuvant Gemcitabine/Capecitabine	Recruiting
PANACHE01-PRODIGE48 (50)	R	168	mFOLFIRINOX + Surgery + adjuvant chemotherapy **versus** FOLFOX + Surgery + adjuvant chemotherapy **versus** Surgery + adjuvant chemotherapy	Recruiting
ALLIANCE A021806	R	352	mFOLFIRINOX + Surgery **versus** Surgery + adjuvant mFOLFIRINOX	Recruiting
PREOPANC-2 (51)	R/BR	375	FOLFIRINOX + Surgery **versus** Gemcitabine + CRT (Gemcitabine + 36 Gy RT) + Surgery + adjuvant Gemcitabine	Completed
PANDAS-PRODIGE- 44	BR	90	mFOLFIRINOX + CRT (capecitabine + 50.4 Gy RT) + Surgery + adjuvant Gemcitabine (or LV5FU) **versus** mFOLFIRINOX + Surgery + adjuvant Gemcitabine (or LV5FU)	Recruiting

R, resectable; BR, borderline resectable; FOLFIRINOX, oxaliplatin, irinotecan, fluorouracil, and leucovorin; mFOLFIRINOX, modified regimen of oxaliplatin, leucovorin, irinotecan, and fluorouracil; chemotherapy, choice at discretion of medical team; LV5FU, infused 5-fluorouracil/folinic acid.

Yet, no phase III trial has compared neoadjuvant chemotherapy with neoadjuvant chemoradiotherapy. Currently, two RCTs are completed or still recruiting patients to answer this question; the PREOPANC-2 trial (51) and the PANDAS-PRODIGE 44 trial (NCT02676349). The first results of the PREOPANC-2 trial are expected by the end of 2022.

In conclusion, the evidence reported so far suggests that chemotherapy should at least be part of the neoadjuvant approach. With a median overall survival that is reported to be longer as compared to RCTs performed with chemoradiotherapy (gemcitabine-based), RCTs on the efficacy of modern chemotherapeutic regimens, with or without concurrent radiotherapy, are warranted.

### New Developments in Neoadjuvant Radiotherapy

The role of neoadjuvant radiotherapy is not yet fully understood. It is likely that radiotherapy results in more R0 resections, fewer lymph-node metastases, and lower locoregional recurrence rates (33, 34, 37). Traditionally, radiotherapy for pancreatic cancer was delivered using conventional linear accelerators using long treatment schedules with low radiation doses per fraction. Currently, an emerging technique is stereotactic radiotherapy in which the tumor is irradiated with high precision to a high dose while sparing the organs surrounding the tumor. The introduction of MR-guided radiotherapy resulted in further development of treatment delivery with an MRI accelerator. Due to good soft tissue visualization using the MRI, combined with plan adaptation during each fraction, even higher doses to the tumor can be safely applied compared with conventional stereotactic radiotherapy (53, 54).

In patients with locally advanced pancreatic cancer, improved outcomes in terms of overall survival and local recurrences were demonstrated in patients receiving higher radiation doses (Biological Equivalent Dose >70 Gy) (55). The recent epidemiological study of Xiang et al. showed that neoadjuvant chemotherapy with stereotactic radiotherapy for patients with RPC is associated with more favorable survival and pathological outcomes than neoadjuvant chemotherapy only or neoadjuvant chemotherapy with conventional radiotherapy (56).

In the randomized phase II ALLIANCE A021501 trial, neoadjuvant chemotherapy followed by stereotactic radiotherapy has been investigated. In this trial, patients with BRPC were randomized between neoadjuvant extended chemotherapy (eight cycles of modified FOLFIRINOX) versus chemotherapy (seven cycles of modified FOLFIRINOX) followed by stereotactic radiotherapy (33–40 Gy in 5 fractions) or hypofractionated radiotherapy (25 Gy in 5 fractions) (57). Recently, the results of an interim analysis were presented after enrollment of 126 patients. The median overall was 31 months after the modified FOLFIRINOX versus 17.1 months after modified FOLFIRNOX followed by stereotactic radiotherapy, though this difference was not significant (58). Since the final article is not yet published, questions remain about the compliance to the treatment and the exact radiation dose, and the methodology of the study. Hence, no firm conclusions can be drawn.

One other trial investigating the combination of neoadjuvant chemotherapy and stereotactic radiotherapy is the BRPCNCC-1 phase II trial, randomizing patients with BRPC between neoadjuvant Gemcitabine/Nab-paclitaxel versus neoadjuvant Gemcitabine/Nab-paclitaxel with stereotactic radiotherapy or neoadjuvant S-1 plus nab-paclitaxel with stereotactic radiotherapy. The dose of radiotherapy is 7.5–8 Gy per fraction, for 5 fractions (59). Currently, this study is enrolling patients. There is a need for more well-planned RCTs to evaluate the efficacy of the combination of neoadjuvant chemotherapy with stereotactic radiotherapy for both RPC and BRPC, since reviews do suggest a benefit of this combined modality (56).

The definitive results of the ALLIANCE 021501 trial and BRPCNCC-1 trial are awaited.

In addition to stereotactic radiotherapy, the use of proton therapy in pancreatic cancer is not clear yet. Proton therapy can result in a sharp dose reduction behind the PTV, thereby reducing the dose to the OARs. However, these sharp dose gradients lead to great challenges in case of motion or position uncertainties. In particle therapy, such as carbon ion or muon, this is also the case. The use of muons, low-energy high-intensity particle therapy, on the other hand, is promising since this therapy results in high linear energy transfer (LET) radiotherapy, with a high biological effectiveness compared to photon or proton radiotherapy. This high-LET radiotherapy is characterized by a reduced oxygen enhancement ratio, leading to high efficacy in hypoxic tumors such as pancreatic cancer (60).

## Future Perspectives

Although improvements in survival have been observed in recent trials, a significant proportion of patients still develop progression during or soon after neoadjuvant treatment. Hence, tools for improving treatment effects are warranted.

Since pancreatic tumors are known to be relatively resistant to chemotherapy and radiotherapy, due to dense stroma causing hypoxia, a complete pathological response has been reported in only 2.5%–7% of the patients (61–63). This hypoxia gives rise to changes in tumor cells, activated by transcription factors such as hypoxia-inducible factors (HIFs). This hypoxia and HIFs can result in not only drug resistance for chemotherapy, but also suppression of cell apoptosis and inhibition of DNA damage (64). One way to reduce this resistance might be by using hyperthermia. With hyperthermia, the temperature of the tumor is raised to 40─44°C for 1 h. Hyperthermia blocks the DNA repair and reduces tumor hypoxia, by increasing the blood flow and the permeability of the blood vessels, resulting in enhanced tumor-cell killing effects of radiotherapy and chemotherapy (65). Adding hyperthermia to chemotherapy and/or radiotherapy is suggested to improve response rate and overall survival in patients with metastatic or irresectable PDAC, without enhancing toxicity (66). The HEATPAC trial is currently accruing patients to investigate the additional role of hyperthermia in patients with locally advanced pancreatic cancer (67). Since the hypoxia and dense stroma are also prominent in patients with resectable or borderline-resectable PDAC, such improved outcomes may also be found in these patient groups.

Second, molecular profiling at the time of diagnosis may guide the selection of individualized systemic treatment. The prospective phase II trial of Tsai et al. found that of the 130 patients with RPC or BRPC, more than 80% of the patients received their neoadjuvant treatment and surgery when molecular profiling was done before start of the therapy (68). Such high resectability rates are unique in neoadjuvant trials and should be tested in a larger randomized trial.

Third, a better understanding of the PDAC immune environment may help to select new treatments to improve outcomes. The effect of immunotherapy in pancreatic cancer has been disappointing so far, probably due to the dense fibrotic stroma and immunosuppressive tumor microenvironment, and the low incidence of deficiency in mismatch repair pathways and subsequent microsatellite instability (69). There is a suggestion that the combination of chemotherapy and/or radiotherapy, combined with immunotherapy may stimulate the immune effect of checkpoint inhibitors in otherwise checkpoint inhibitor-resistant cancers. The role of combined immune therapy (pembrolizumab, tremelimumab, and ipilimumab) or vaccine with chemo(radio)therapy seems to result in a synergistic effect with an increased overall survival found for patients with localized disease (70).

## Conclusions

There is sufficient evidence from randomized trials to conclude that neoadjuvant treatment results in better outcome than immediate surgery, particularly in patients with BRPC. For RPC the evidence is accumulating. So far, all randomized evidence is based on gemcitabine-based chemo(radio)therapy schedules. Radiotherapy appears to play a role, particularly in pathological endpoints (i.e., pathological lymph nodes and resection margin) and local control. Whether neoadjuvant FOLFIRINOX or Gemcitabine/Nab-paclitaxel is better than conventional chemoradiotherapy remains to be proven, as well as the role of radiotherapy in combination with these chemotherapy schedules. Future research should further explore on combinations of modern systemic therapies combined with (for instance, stereotactic) radiotherapy, and chemotherapy-enhancing treatment modalities, such as immunotherapy and hyperthermia.

## Author Contributions

EV and GvT wrote the first draft of the manuscript. All authors contributed to manuscript revision, read, and approved the submitted version.

## Conflict of Interest

JV-G has served as a consultant for Amgen, AstraZeneca, MSD, Pierre Fabre, and Servier, and has received institutional research funding from Servier (outside the submitted work). Johanna Wilmink: Research funding: Servier, Halozyme, Novartis, Celgene, Astra Zeneca, Pfizer, Roche, Merck, and Servier; Consulting/advisory role: Shire, Celgene, Servier, and Merck/MS (outside the submitted work).

The remaining authors declare that the research was conducted in the absence of any commercial or financial relationships that could be construed as a potential conflict of interest.

## Publisher’s Note

All claims expressed in this article are solely those of the authors and do not necessarily represent those of their affiliated organizations, or those of the publisher, the editors and the reviewers. Any product that may be evaluated in this article, or claim that may be made by its manufacturer, is not guaranteed or endorsed by the publisher.
